# Anterior inferior tibiofibular ligament avulsion fractures in operatively treated ankle fractures: a retrospective analysis

**DOI:** 10.1007/s00402-019-03138-2

**Published:** 2019-02-15

**Authors:** Merel F. N. Birnie, Kaz L. J. van Schilt, Fay R. K. Sanders, Peter Kloen, Tim Schepers

**Affiliations:** 1 Trauma unit, Amsterdam UMC, Location AMC, Meibergdreef 9, 1105 AZ Amsterdam, The Netherlands; 2 Department of Orthopedic surgery, Amsterdam UMC, Location AMC, Amsterdam, The Netherlands

**Keywords:** Ankle fracture, Wagstaffe, Syndesmosis, Fixation

## Abstract

**Background:**

There is no consensus about the optimal treatment of anterior inferior ligament avulsion fractures of the ankle. The aim of this study is to provide insights regarding the incidence of anterior inferior ligament avulsion fractures, the association with fracture type, and correlation with treatment.

**Methods:**

This study is a retrospective analysis in a level-1 trauma center of adult patients with an ankle fracture operated between the dates 01-01-2009 and 01-09-2017 who had a pre- and postoperative CT-scan. Within the study population, the incidence of AITFL avulsion fracture was defined. Primary outcome was the type of avulsion fracture and related treatment. Secondary outcome was additional surgery in relation to the initial treatment.

**Results:**

In total, 65 of 252 (25.8%) patients were diagnosed with an anterior inferior ligament avulsion fracture. Zero patients had a Wagstaffe type 1 fracture, 28 (43.1%) had a type 2, 32 (49.2%) had a type 3, and 5 (7.7%) had a type 4. There was a correlation between Wagstaffe type 2 and Weber B fractures, *p* < 0.0001, and Wagstaffe type 3 avulsions were correlated with a Weber C fracture, *p* < 0.0001. Thirty-five of the avulsed fragments (53.8%) were smaller than 5 mm. In 13 (20%) of patients with anterior inferior ligament avulsion fracture, the avulsed fragments were directly fixated during initial surgery. Size and direct fixation of the fragment were significantly correlated (*p* < 0.0001). Within the anterior inferior ligament avulsion fracture group, only a total of four patients (6.2%) underwent a revision.

**Conclusion:**

In the current study, an incidence of 25.8% of anterior inferior ligament avulsion fracture in surgically treated ankle fractures is reported. A correlation between the type of Wagstaffe injury and Weber classification was showed. Most fragments smaller than 5 mm were not fixated; however, not all injuries needed syndesmotic screws due to syndesmotic instability.

**Level of evidence:**

Level IV.

## Introduction

The anterior inferior tibiofibular ligament (AITFL) is one of the ligaments that stabilize the tibiofibular syndesmosis, contributing to ankle stability [1]. An unstable syndesmosis requires restoration of congruity, since this is one of the main prerequisites of good-long-term functional outcome [2].

The current literature shows an incidence of syndesmotic injuries of 10% in ankle fractures overall, up to 20% in ankle fractures requiring surgery [3]. Insufficiently treated syndesmotic injury will result in chronic ankle instability and posttraumatic osteoarthritis [4]. Technical aspects, postoperative policy, and alternatives of the syndesmotic screw are still widely discussed in literature [5–8].

Because of the position of the AITFL, the ligament could be at risk in trauma mechanisms involving external rotation. The external rotation forces during injury may lead to rupture of the ligament or an avulsion fracture either at the side of the fibula or the tibia as described by Lauge-Hansen [9–11].

In 1875, Wagstaffe described avulsed fragments of the fibula for the first time [12]. Later, in 1886, LeFort described the same vertical fracture of the anteromedial portion of the fibula (Wagstaffe tubercle) at the site of the anterior tibiofibular ligament, which is nowadays called the “Le Fort–Wagstaffe fracture” [13, 14].

A fracture of the anterolateral tibial epiphysis or “Tillaux fracture” is most commonly seen in adolescents [15–17]. Paul Jules Tillaux was the first to describe this type of an avulsion fracture of the lateral tibia in 1892, after experimenting on cadavers. A similar injury of the anterolateral tibia was later described by Chaput and has since been called the fracture of Tillaux–Chaput [14, 18].

**Fig. 1:**
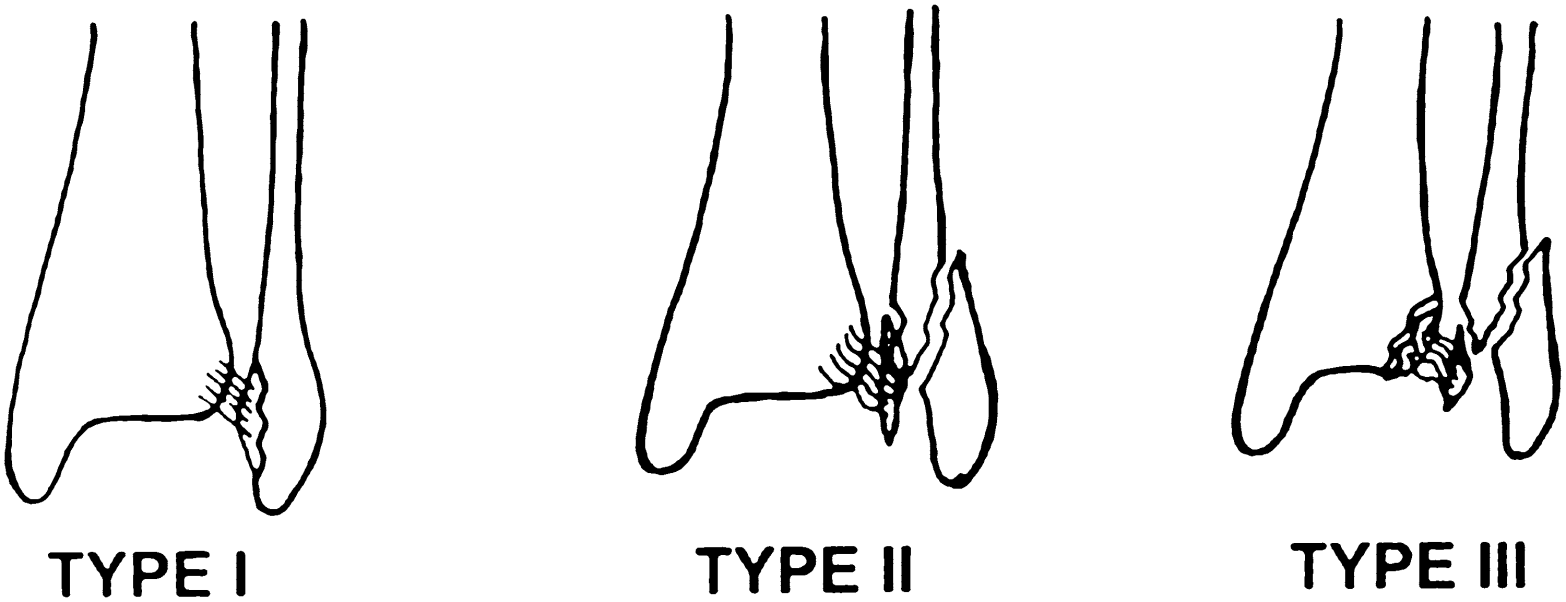
AITFL avulsion fracture classification

Between 1970 and 2012, three case series have been published, investigating the incidence and treatment of AITFL avulsion fractures [19–21]. The incidence of AITFL avulsion fractures ranged from 10 to 12.4% and was subdivided according to the Wagstaffe classification system (Fig. 1) [21].

Currently, there is no consensus about the optimal treatment of AITFL fractures [20, 21]. The aim of this study was to provide an overview of the incidence of AITFS avulsion fractures, the associated fracture classification, treatment, and revision rate. Second, we investigated the need for revision depending on fixation of the avulsed fragment.

## Materials and methods

This was a retrospective study of patients with ankle fractures admitted in a level-1 trauma center between 01-01-2009 and 01-09-2017. A search was performed in the electronic patient database, including all patients with a specific operation code associated with the surgical treatment of a malleolar ankle fracture. Whether or not operative treatment would be necessary was decided by one of the five trauma surgeons involved in the study, based on the national guidelines focused mainly on stability of the fractured ankle [22]. Patients were excluded (1) if they were below the age of 18, (2) if a pre- and postoperative CT-scan was not performed, (3) in case of a pilon fracture, (4); if the initial surgery took place in a different center, or (5) if they objected to the use of their medical records. Because of the retrospective design of this study, no approval from the Internal Reviewing Board was necessary.

Baseline characteristics such as age at time of surgery, gender, medical history, medication use, ASA classification, smoking history, and side of injury were obtained from the medical records.

All ankle fractures were classified by the attending trauma surgeon according to the Lauge-Hansen [9, 10], Weber [23, 24] and Pott’s [25] classification system, using the pre- and postoperative radiographic images such as the CT-scans and X-rays of the injured ankle. All fractures were re-classified for study purposes by a Foot–Ankle specialized trauma surgeon. The Pott’s system counts the number of fractured malleoli in an ankle fracture with a maximum of three involved malleoli (medial malleolus, lateral malleolus, and the posterior malleolus). The Weber and Lauge-Hansen system were combined in this study to classify the ankle fractures [26].

After classification, the presence of AITFL avulsion fractures was determined by two observers using the preoperative CT-scan and classified according to a modificatied Wagstaffe classification [21]. Subsequently, based on the preoperative CT-scan, the fragments were measured at their widest point and divided into two groups; (1) fragments smaller than 5 mm and (2) fragments of 5 mm or larger, considering that a fragment smaller than 5 mm would be too small to fixate directly using screw fixation.

Treatment including type of fixation of the avulsed fragment was recorded. Furthermore, the placement and type of syndesmotic screws was recorded.

Finally, the need for additional surgery (e.g., revision osteosynthesis, arthroscopy, and postoperative wound infections) was registered. Only revision osteosynthesis and arthroscopy were labeled as revisions related to the treatment of the avulsed fragment during surgery.

### Statistical analysis

All data processing and statistical analysis was performed using IBM^®^ Statistical Package for the Social Sciences (SPSS^®^), version 24. Differences in baseline characteristics between patients with an AITFL avulsion fracture and without an AITFL avulsion fracture were analyzed using an independent samples *T* test for continuous data and the Pearson-chi square for binary data. Within the study population, the incidence of AITFL avulsion fractures was defined. To investigate the correlation between the type of ankle fracture and the type of AITFL avulsion fracture, 2 × 2 contingency tables were created and significance were assessed using the Pearson-Chi-square test was used. Differences between revisions in the AITFL avulsion fracture group and the non-AITFL avulsion fracture group have been analyzed using either the Fishers exact test or the Pearson-Chi-square test, depending on the sample size. Only revision surgery was included in statistical analysis. *p* values lower than 0.05 were considered statistically significant.

## Results

A total of 670 patients with operatively treated ankle fractures were extracted from the electronic database. Based on the exclusion criteria, 418 patients were removed from further analysis. As a result, 252 patients were found eligible for this study. The mean age at time of surgery was 45 (SD 16.2) years, 52% (131) of these patients were male, and most patients had an ankle fracture on the right side (58.3%). The mean follow-up was 60.5 months, ranging from 5 to 107 months.

**Fig. 2:**
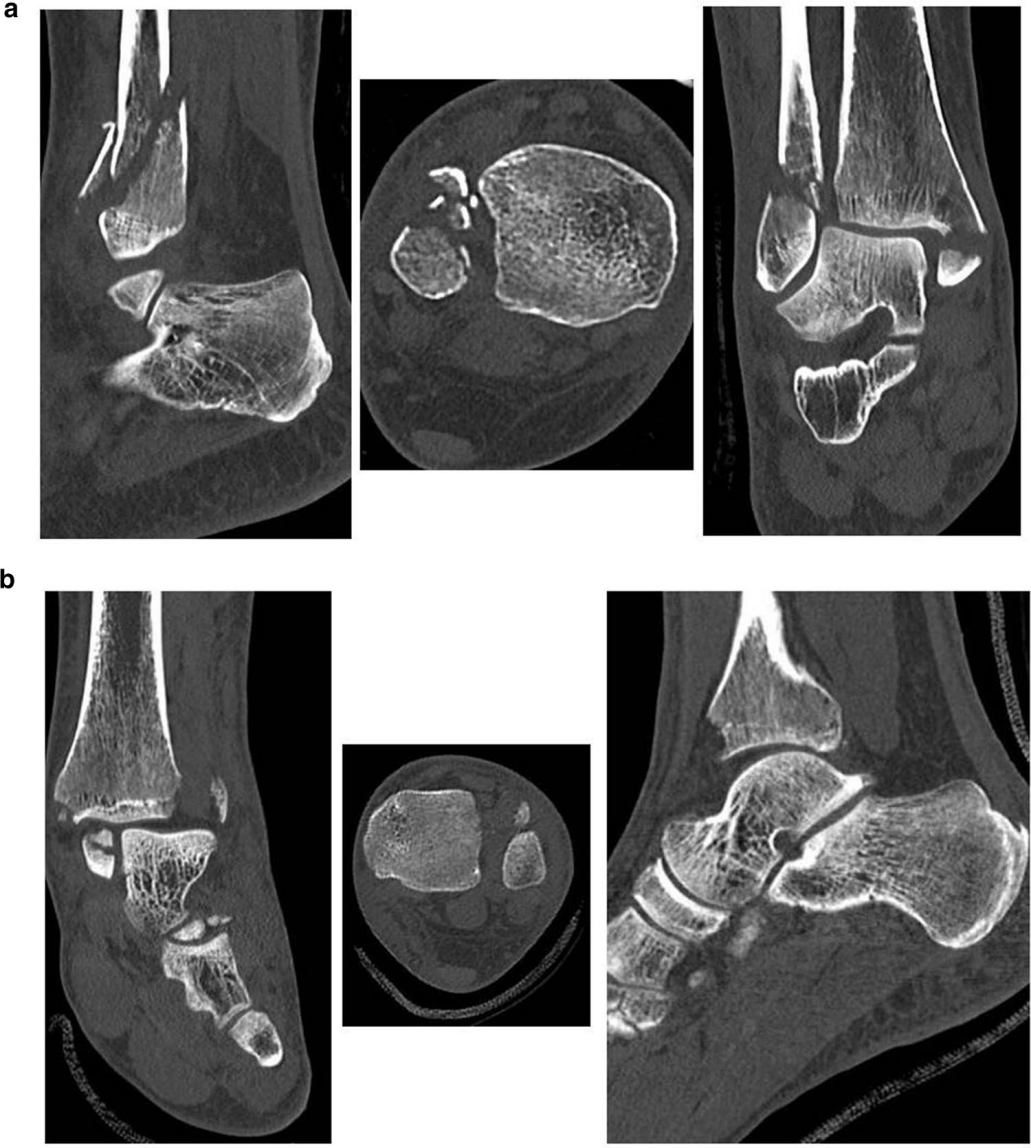
**a** Wagstaffe type II fracture. **b** Wagstaffe type III fracture

Patients were divided into two groups: the group with an anterior inferior tibiofibular ligament avulsion fracture (AITFL avulsion fracture) and the group without an avulsion fracture (no AITFL avulsion fracture). No statistical differences between the groups were observed with regards to baseline characteristics and Weber classification (Tables 1, 2). Sixty-five (25.8%) patients were diagnosed with an AITFL avulsion fracture. Of these 65 patients; zero patients had a Wagstaffe type 1 fracture, 28 (43.1%) had a type 2, 32 (49.2%) had a type 3, and 5 (7.7%) had a type 4 (Fig. 2). Regarding the size of the avulsed fragments, 35 (53.8%) were smaller than 5 mm and 30 (46.2%) were 5 mm or larger.

**Table 1:** Patient characteristics

Patient characteristics total (*n* = 252)	No AITFL avulsion fracture (*n* = 187)	AITFL avulsion fracture (*n* = 65)	
Mean age (years)	43.4 (SD 15.2)	49.4 (SD 18.0)	*p* = 0.93
Sex			*p* = 0.167
Male	102 (54.5%)	29 (44.6%)	
Female	85 (45.5%)	36 (55.4%)	
Side of ankle fracture			*p* = 0.789
Left	77 (41.2%)	28 (43.1%)	
Right	110 (58.8%)	37 (56.9%)	
History of DM	12 (6.5%)	2 (3.1%)	*p* = 0.318
History of CVD	26 (14%)	12 (18.5%)	*p* = 0.359
History of smoking	46 (24.6%)	12 (18.5%)	*p* = 0.752
Mean packyears (years)	20.1 (SD 12.7)	35.0 (17.3)	*p* = 0.803
ASA classification			*p* = 0.462
1 and 2	168 (89.8%)	60 (92.3%)	
3 and 4	7 (3.7%)	4 (6.2%)	
Missing	12 (6.4%)	1 (1.5%)	

**Table 2:** Classification

Classification system	Total (*n* = 252)	No AITFL avulsion fracture (*n* = 187)	AITFL avulsion fracture (*n* = 65)	
Weber				
A (SAD)	2 (0.8%)	2 (1.1%)	0	*p* = 0.999
B (SER)	156 (61.9%)	117 (62.6%)	39 (60.0%)	
C (PER)	91 (36.1%)	67 (35.8%)	24 (36.9%)	*p* = 0.811
Missing	3 (1.2%)	1 (0.5%)	2 (3.1%)	*p* = 0.811
Pott’s				
Unimalleolar	64 (25.4%)	56 (29.9%)	8 (12.3%)	*p* = 0.005
Bimalleolar	65 (25.8%)	50 (26.7%)	15 (23.1%)	*p* = 0.561
Trimalleolar	123 (48.8%)	81 (43.3%)	42 (64.6%)	*p* = 0.003

Table 2 shows de Weber and Pott’s classification of the ankle fractures for the no AITFL avulsion fracture and the AITFL avulsion fracture group. In three of the patients, classification according to Weber was not possible. In the no AITFL avulsion fracture group, one of them had an isolated posterior malleolar fracture, and in the group with AITFL avulsion fracture, there were two patients with isolated Tillaux-Chaput fractures.

The presence of an AITFL avulsion fracture was associated with the occurrence of a trimalleolar ankle fracture, *p* = 0.003 (Table 2).

Furthermore, there was no significant correlation between the presence of AITFL avulsion fracture in general and a specific Weber fracture type. However, there was a significant correlation between Wagstaffe type 2 and Weber B fracture specifically (*p* < 0.0001), and Wagstaffe type 3 avulsions were correlated with a Weber C fracture (*p* < 0.0001) (Table 3).

**Table 3:** Weber type and Wagstaffe classification

	Total	Type 1	Type 2	Type 3	Type 4
Weber A (SAD)	0	0	0	0	0
Weber B (SER)	39	0	27 (69.2%)	9 (23.1%)	3 (7.7%)
Weber C (PER)	24	0	1 (4.2%)	21 (87.5%)	2 (8.3%)
Total	63	0	28	30	5

Of the 65 patients with an AITFL avulsion fracture, 42 patients of them received some form of fixation. The AITFL avulsion fracture was fixated using direct fixation of the fragment in 13 (20%), the placement of a syndesmotic screw (indirect fixation) in 17 (26.2%), or a combination of direct fixation and placement of a syndesmotic screw in 12 (18.5%) (Table 4).

**Table 4:** Fixation

	< 5 mm	> 5 mm	
No fixation	16 (45.7%)	7 (23.3%)	*p* = 0.060
Fixation of fragment (direct fixation)	1 (2.9%)	12 (40.0%)	*p* < 0.0001
Syndesmotic screw (indirect fixation)	12 (34.3%)	5 (16.7%)	*p* = 0.107
Both	6 (17.1%)	6 (20.0%)	*p* = 0.767
Total	35	30	65

Various ways of direct fixation of the fragment were performed; in 11 cases with a single screw, in four cases with two screws, in four cases with a plate and screws, in four with a bone-anchor, in one case with Zuggurtung fixation, and in one with a ligament suture repair.

Direct fixation was significantly correlated (*p* < 0.0001) with the size of the fragment > 5 mm (Table 4).

A total of 37 (14.7%) patients from the entire study population (*n* = 252) underwent an additional surgery. Sixteen (6.3%) patients needed surgery because of postoperative wound infection. In 15 patients, revision surgery was performed, due to malreduction (*n* = 5), secondary dislocation (*n* = 5), mal-union (*n* = 2), or non-union (*n* = 3). Other additional surgeries were performed in six patients. Four of these patients underwent ankle arthroscopy for impingement, one underwent an ankle arthrodesis due to posttraumatic osteoarthritis, and one developed a compartment syndrome.

In the AITFL avulsion fracture group, a total of four patients (6.2%) of the 65 patients underwent an additional surgical procedure. All of these patients belonged to the group were the fragment was not directly fixated, but no statistically significant difference was observed compared to the group with direct fixation of the avulsed fragment. Three patients needed re-fixation. One patient underwent an arthroscopy with removal of loose bodies due to anterior impingement complaints, 1 year after the initial surgery. This concerned a 43-year-old woman with a trimalleolar Weber B ankle fracture, with a Wagstaffe type 2 fragment smaller than 5 mm. Neither the fragment itself, nor the syndesmosis was fixated initially.

## Discussion

In the present study, investigating patients who underwent surgery due to a malleolar ankle fracture, a high incidence (25.8%) of AITFL avulsion fractures was observed in comparison with the previous literature. Park et al. [21] reported an incidence of 12.4%. Selection bias could be a possible explanation for this difference in incidence. Were Park and al. only used the standard X-rays to identify AITFL avulsion fractures, this study included only patients with a pre- and postoperative CT-scan, making it easier to identify avulsed fragments. Furthermore, this study reported on patients who were treated in a level-1 trauma center, where generally more “complex” ankle fractures are treated.

Just like in the other studies, no Wagstaffe type 1 avulsion fractures were observed [19, 20]. Historically, only Wagstaffe himself reported the type 1 fracture, an isolated avulsion fracture of the anterior tubercle of the distal fibula [12]. Interestingly, he suspected the existence of this type of avulsion fracture after only physical examination in a time where no X-rays were available. The existence of this fracture type of the distal fibula is questionable, considering the absence of reliable proof and no additional described cases in literature.

On the other hand, we evaluated only operative treated ankles, as CT-scans are mostly not performed in conservative-treated ankles. Wagstaffe type 1 fractures might be underreported, since they could easily be missed on a conventional X-ray.

Park et al. only observed AITFL avulsion fractures in patients with a Weber B type ankle fracture. In contrast, this study also had a proportion of patients with Weber C fractures in the AITFL avulsion fracture group (30.6%). This is the first study which shows avulsion fractures of the AITFL which can occur with the foot in both supinated and pronated position during external rotational forces.

In the previous series, the majority of cases with an AITFL avulsion fracture concerned Wagstaffe type 2 fractures. In this study, we observed a more equal distribution between Wagstaffe type 2 (43.1%) and type 3 fractures (49.2%). Like other studies, a correlation was reported between Wagstaffe type 2 avulsion fractures and Weber B type ankle fractures. However, this study also correlated the Wagstaffe type 3 (Tillaux–Chaput) fracture with Weber C type ankle fractures. With the numbers available, a significant correlation, which was found in this study, could be indicative of a hypothesis that there is more tension on the insertion of the AITFL on the Wagstaffe tubercle with the foot in supinated position, and on the Chaput tubercle during pronated position.

It seems that fragment size has an influence on the way of fixation of the avulsed fragment. Recently, Diallo et al. [27] presented a case series in which they described screw fixation of ten avulsion fractures, sized 5 mm or larger. This corresponds with the results of this study, in which we observed a significant correlation in fragment fixation (direct fixation) of fragments of 5 mm or larger. This study suggests that the size of an avulsed fragment determines the way of fixation.

Haraguchi et al. described that 35% of the conservatively treated avulsion fractures did not heal, without mentioning fragment size [28]. The correlation between size of the avulsed fragments and type of fixation has not been mentioned before in the literature. Park et al. fixated all the fragments with non-absorbable sutures through anchoring holes made by Kirschner wires. It is conceivable that this technique has its limitations in smaller fragments. In the series of Chung et al. [20] in 2012, most of the avulsed fragments were fixated with mini screws or Kirschner wires, while the others were repaired with a ligament suture. A variety of types of fixation of the avulsed fractures were mentioned in this study. The fixation with a single screw was most common.

**Fig. 3:**
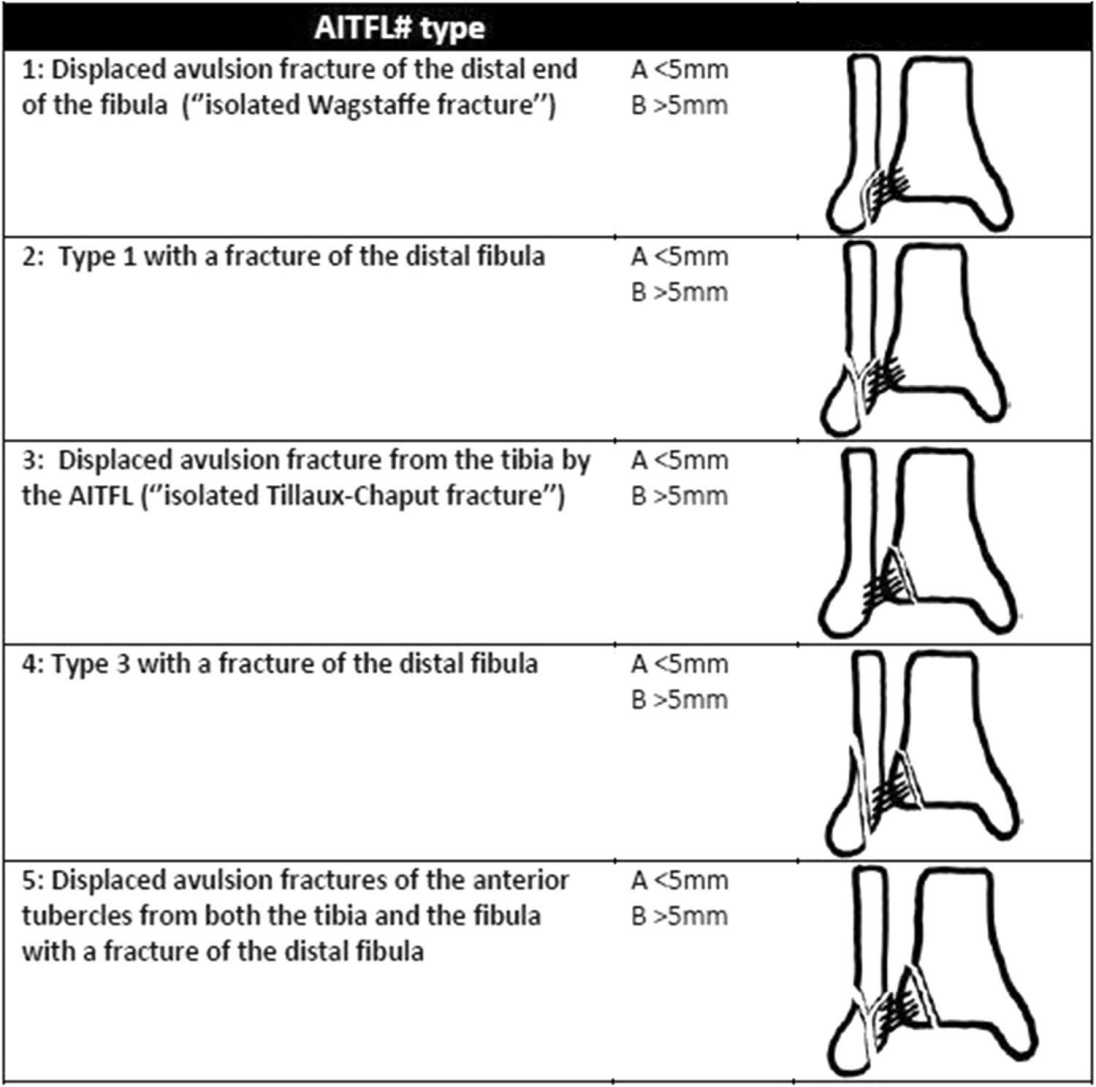
Modified AITFL avulsion fracture classification system

Due to the fact that direct fixation of an avulsed fragment appeared to be dependent on the size of the fragment, we would like to propose a new AITFL avulsion fracture classification system (Fig. 3). We adjusted the classification for this variable into the subtypes: (a) < than 5 mm in diameter and (b) ≥ 5 mm in diameter. Avulsion fractures of subtype b should be taken into consideration for fixation. Furthermore, we added an additional type of avulsion fracture: the isolated avulsion fracture of the anterolateral distal tubercle of the tibia (isolated Tillaux–Chaput fracture). This type of fracture has not been frequently reported in the previous study and was observed twice in this study.

Due a low number of revisions (*n* = 4) in the AITFL avulsion fracture group, no significance could be observed between the groups with non-direct fixated fragment versus direct fixated fragments. However, all four complications did occur in the non-fixated group. In the three cases which underwent revision surgery, a syndesmotic screw was placed during the initial surgical procedure, which should have stabilized the ankle. It is questionable whether direct fixation of the fragment would have prevented a revision procedure. Other variables, like smoking or osteoporosis, could have affected the healing of the bone as well [29, 30]. In patients with secondary dislocation premature mobilization could not be ruled out. Anterior impingement syndrome is a complication which was also seen in one case in the study by Park et al. [21].

This study only reports on the incidence of AITFL avulsion fractures in patients who had an indication for surgery for their ankle fracture and underwent a CT-scan. Due to the retrospective nature of this study, the optimal treatment of AITFL avulsion fractures accompanied by ankle fractures can only be suggested.

Finally, this study does not incorporate the results of functional outcome, which could have given a clue into the direction whether it is important to fixate the fragment. However, in retrospective studies, it is not possible to include surveys such as Olerud–Molander Ankle Scores (OMAS) [31], unless they were part of the standardized postoperative procedure at fixed times.

In conclusion, the current study reports a CT-scan incidence of 25.8% of AITFL avulsion fracture in surgically treated ankle fractures. Significant correlations with Weber B/C type ankle fractures and Wagstaffe type 2/type 3 fractures were found. Most fragments larger than 5 mm were fixated. Based on the size of the avulsed fragments and possibilities of fixation, a new AITFL avulsion fracture classification system has been proposed.
